# Biosilicate® Ototoxicity and Vestibulotoxicity evaluation in guinea-pigs

**DOI:** 10.1016/S1808-8694(15)30515-2

**Published:** 2015-10-18

**Authors:** Eduardo Tanaka Massuda, Lucas Lisboa Maldonado, Jessé Teixeira de Lima, Oscar Peitl, Miguel Ângelo Hyppolito, José Antonio Apparecido de Oliveira

**Affiliations:** 1 PhD, Assistant Professor - Department of Ophthalmology, ENT and Head and Neck Surgery of the Ribeirão Preto Medical School - University of São Paulo (USP); 2 ENT Resident Physician – Department of Ophthalmology, ENT and Head and Neck Surgery of the Ribeirão Preto Medical School - University of São Paulo (USP; 3 ENT Resident Physician – Department of Ophthalmology, ENT and Head and Neck Surgery of the Ribeirão Preto Medical School - University of São Paulo (USP; 4 PhD, Professor of Materials Engineering, Federal University of São Carlos; 5 PhD, Assistant Professor - Department of Ophthalmology, ENT and Head and Neck Surgery of the Ribeirão Preto Medical School - University of São Paulo (USP); 6 Full Professor - Department of Ophthalmology, ENT and Head and Neck Surgery of the Ribeirão Preto Medical School - University of São Paulo (USP)

**Keywords:** middle ear, ossicular prosthesis, toxicity

## Abstract

Changes, destructions and interruptions in middle ear ossicular chain architecture may be caused by infection, trauma, tumors, congenital alterations or prior surgeries. Nonetheless, infectious and inflammatory processes, focal or generalized which affect the middle ear are the most prevalent, causing a great demand for ossiculoplasty. Biosilicato® is a new material which can be used in the middle ear with the goal of reconstructing the ossicular chain. It is a bioactive type A vitroceramic, in other words, it binds to bone or soft tissue in a matter of a few hours, thanks to the formation of hydroxy-carbonateapatatie in its contact surface when in contact with body fluids.

**Aims:**

The goal of the present paper is to assess biosilicate ototoxicity and vestibular toxicity in experimental animals, for later use in humans.

**Materials and Methods:**

This a clinical and experimental study in which otoacoustic emissions were performed before and after the placement of Biosilicate in the middle ear of experimental animals and a scanning electron microscopy was carried out in the cochlea, saccule, utriculus and macula of the semicircular canals after 30 and 90 days to assess oto and vestibular toxicity.

**Results:**

There were no signs of oto or vestibular toxicity in any of the groups associated with biosilicate.

**Conclusion:**

Biosilicate is a safe material to be used in ossiculoplasties

## INTRODUCTION

Changes, destructions and interruptions in the middle ear ossicular chain in humans may be of infectious origin, trauma, tumor, congenital or due to prior surgery. Nonetheless, infectious inflammatory processes^1^, focal or generalized which affect the middle ear are the most prevalent. This disease is defined as otitis media.

Otitis media affects 2% of the population, and 8% of school age children. It affects millions of persons every year, generating huge financial costs^2^.

Thus, if we analyze only the number of chronic otitis media and its many variations: cholesteatoma, cholesterol granuloma, adhesive otitis, granulomatous otitis, the percentage of people who may require surgical intervention to correct ossicles defects is enormous. Surgeries for the reconstruction of the ossicular chain are called ossiculoplasties. The first reconstructions were made by Wullstein in 1956^3^.

Different materials are used in ossiculoplasties, all with good results^4^, which according to their own nature and origin are called autografts, homografts and synthetic prosthesis^5^.

The autografts^6^ are the remains of the ossicles, cranial cortical bones^7^ and cartilage of the patient^8^.

The homologous grafts are ossicles obtained from banks of organ donation; however, they are no longer used because of AIDS.

Synthetic materials are the ones most used. Among them we have titanium prosthesis^9^, ^10^, ^11^, ceramic^12^, ^13^, hydroxiapatite^14^, ^15^, plastipore^16^, and bone cement^17^.

Biosilicate® is a new material which can be used in middle ears with the goal of reconstructing the ossicular chain. It is type A bioactive vitroceramic, it binds to the bone tissue or soft tissue in a few hours, because hydroxyapatite forms on its contact surface when in contact with body fluids^18^.

The goal of the present study was to assess biosilicate ototoxicity and vestibulotoxicity in laboratory animals, for later use in humans.

## MATERIALS AND METHODS

According to approval by the Ethics Committee under protocol # 046/2005, we used 15 albino male lab animals weighing between 400g to 600g, with Preyer's reflex present and Distortion Product Otoacoustic Emissions (ILO 92 CAE System otodynamics LTD equipment) present in both sides. These otoemissions were measured in the pre and postoperative (before the animals were slaughtered) without sedation, and then the animals were broken down into 3 groups of 5 animals in each.

These animals were anesthetized with intraperitoneal pentobarbital 40mg/kg and injected with penicillin; immediately after we injected the retroauricular region with 2% xylocaine associated to adrenalin 1:200.000 bilaterally. A 1cm retroauricular incision was made all the way to the bulla, causing bilateral exposure; a bone window was open under microscopic view (DF Vasconcellos Microscope) to access the middle ear.

**Figure 1:**
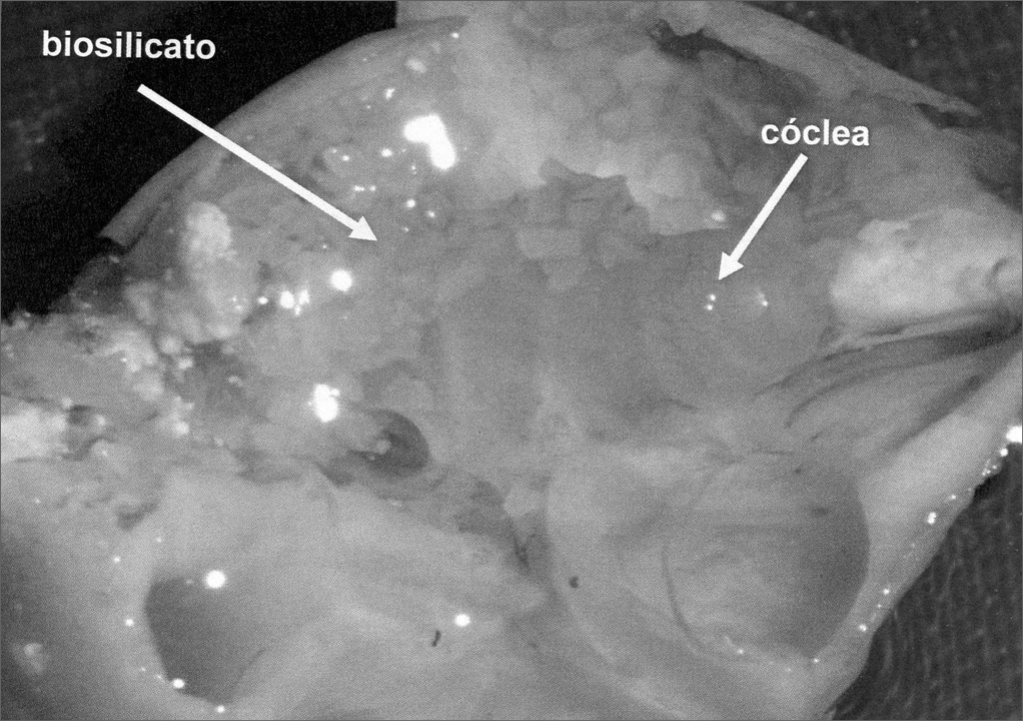
Lab animal cochlea with powder otosilicate around the cochlea and round window.

In groups 1 and 2 we used powder Biosilicate® (Fig. 1), closing the bulla with bone cement in a total of 10 lab animals. In Group 3 (control) the bulla was opened and closed immediately after opening with dental cement.

In Group 1, the animals were slaughtered 30 days later and in Group 2 and 3 they were slaughtered 90 days after the surgical procedure. Their cochleas, saccule, utricle and vestibular ampullae were removed for scanning electron microscopy (SEM).

The animals were slaughtered in a scheduled fashion under anesthesia by ether, and they were later beheaded in order to remove the cochlea from their bullas.

With microscopic dissection the cochleas were soaked in a fixation solution of 3% Glutaraldehyde at 4° Celsius and kept in solution for 24 hours for fixation purposes. The steps that followed were carried out at the Electron Microscopy Lab of the Molecular and Cellular Biology and Pathogenic Bioagents Department.

The 3% glutaraldehyde solution in 0.1M, pH equals; 7.4 phosphate buffer was injected in the cochleas through the round window for 4 hours at 4° Celsius, flushed three times for 5 minutes with the same buffer solution, afterwards they were fixed with 1% osmium tetroxide for 2 hours at 4° Celsius and were submitted to dehydration at room temperature and in an increasing battery of ethanol (50%, 70%, 90% and 95% - once, for 10 minutes in each concentration) and absolute ethanol three times for 15 minutes. After dehydration, it was dried by the CO_2_ critical point method, when the water was taken off the material. After being fixed in a proper specimen holder, the material was coated with gold vapors in a vacuum chamber and examined under SEM (Jeol JSM 5200 microscope).

After photographed, the results obtained from the SEM were analyzed through cochleograms. We counted the number of outer hair cells on the cochlear basal turn in a certain photographic fields and we counted 10 cells, present or absent.

The saccular and utricle maculae, as well as the ampullae of the semi-circular canals were prepared the same way as the cochlea and assembled on a separate specimen holder. We established that the damage to the vestibular neuroepithelium would be based on the disappearance or rarefaction of the stereocilia bundles of the vestibular hair cells assessed by SEM.

## RESULTS

**Figure 2:**
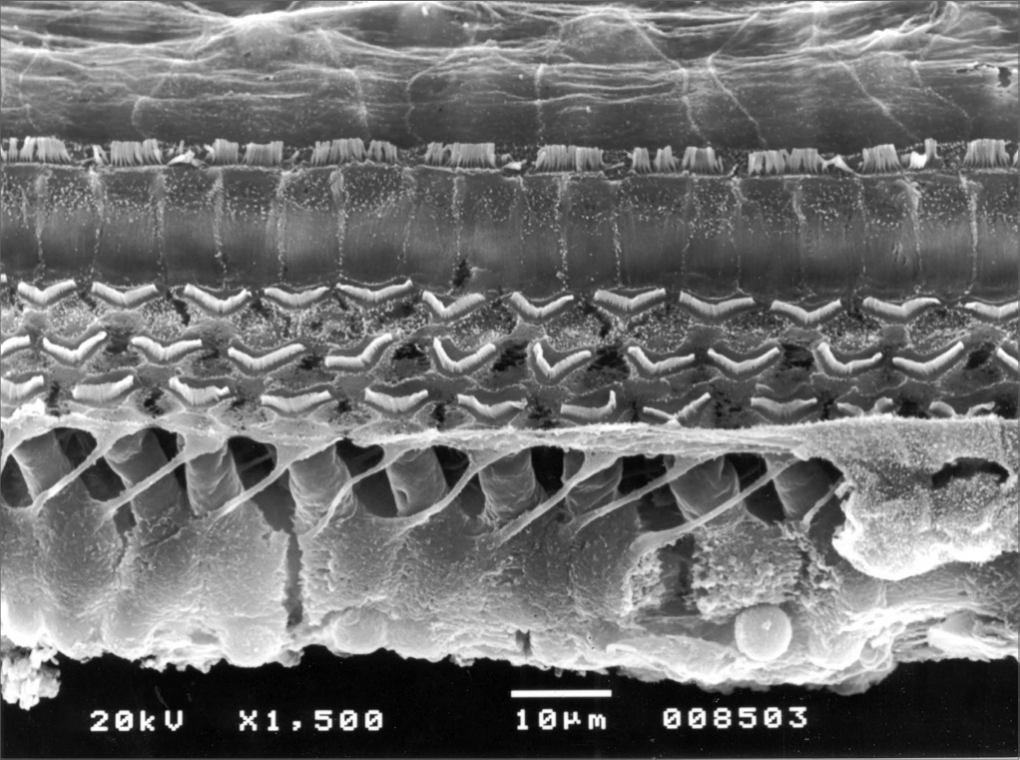
Cochlea basal ramp belonging to an animal from the otosilicate group after 30 days, where we can see all the external and internal hair cells.

All the 5 lab animals (10 ears) which were slaughtered within 30 days presented inner and external hair cells preserved without any sign of ototoxicity (Fig. 2) when assessed by SEM. We carried out the Fisher's exact test (p≤0.05 significance level) with a p=1 result, indicating no ototoxicity in the otosilicate group, statistically significant.

**Figure 3:**
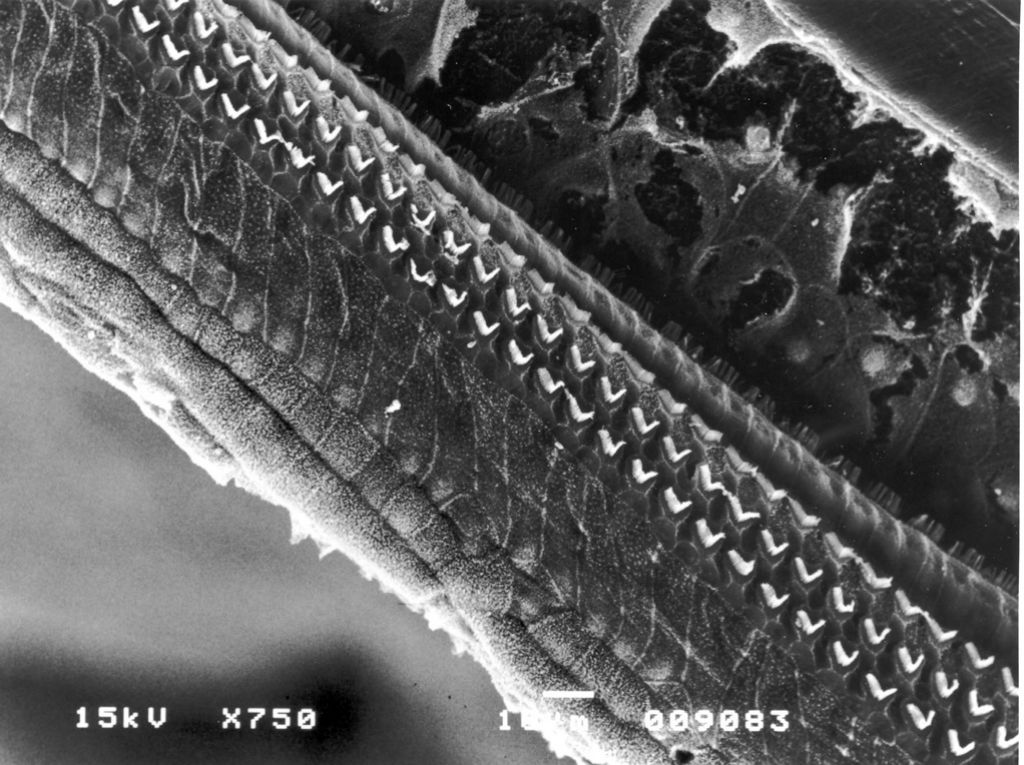
Cochlea basal ramp belonging to an animal from the otosilicate group after 90 days. Inner and outer hair cells without lesion.

The laboratory animals which were slaughtered within 90 days had otosilicate in Group 2, and only bulla cover in Group 3 (5 animals in each group), making up a total of 20 ears, did not show signs of ototoxicity (Fig. 3). Through the Fisher's exact test, p=1, there was no ototoxicity in groups 2 and 3, with statistical significance.

**Figure 4:**
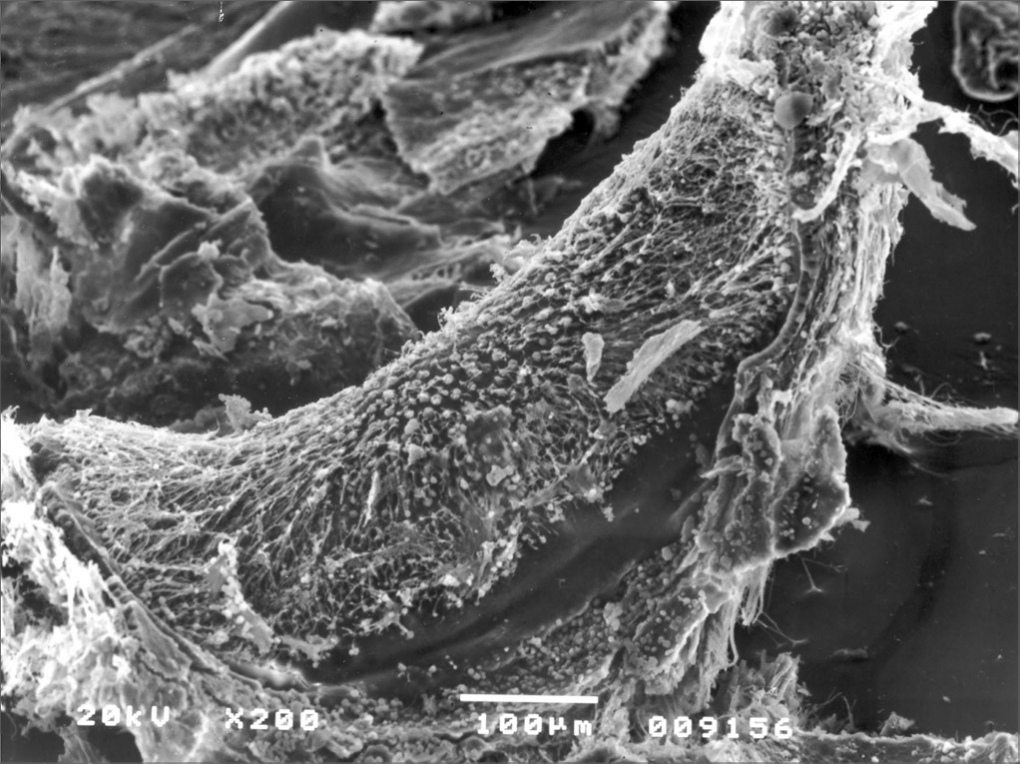
Crest of the lateral canal ampulla without alteration. Otosilicate group – 30 days.

**Figure 5:**
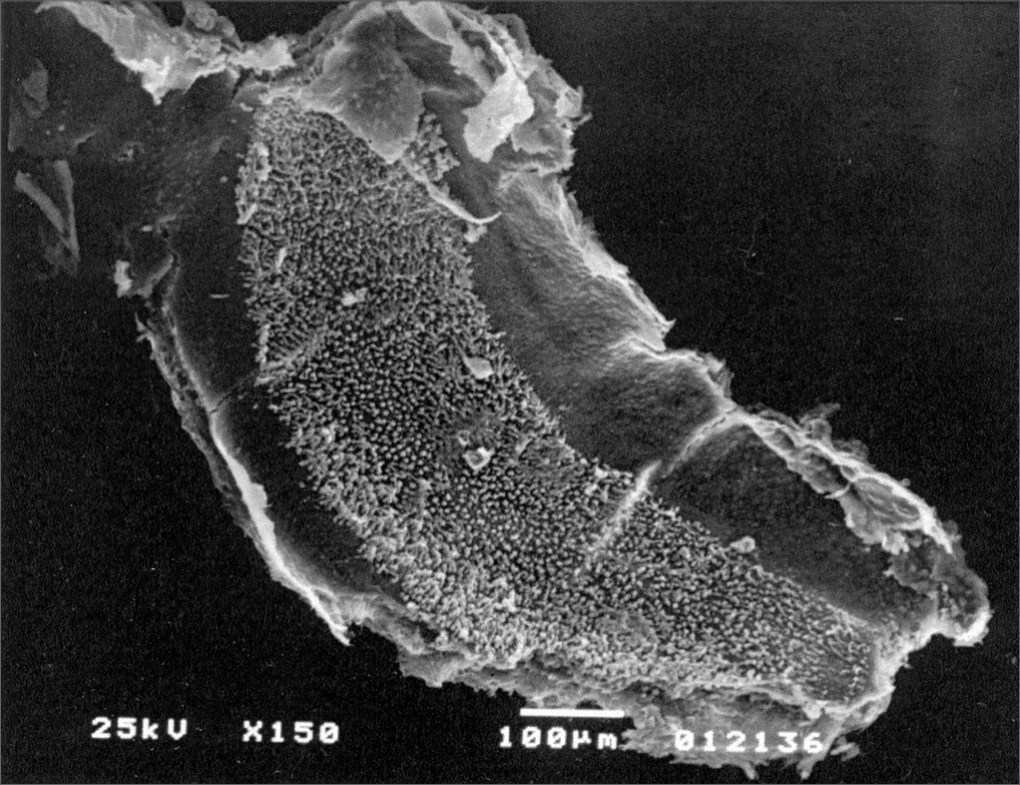
Saccular macula of the otosilicate group – 90 days, without lesion.

We did not observe signs of vestibulotoxicity in the animals tested within 30 and 90 days of exposure to otosilicate (p>0.05). (Figure 4, Figure 5)

## DISCUSSION

Different animal models were tested in order to assess the ototoxicity of biocompatible materials for use as prosthesis in the middle ear such as rats^19^, mice^20^, guinea pigs^21^, rabbits1, cats^22^. Zikk et al.^21^ utilized ceravital grains, not observing hearing loss measured by the brainstem evoked auditory potential in 100% of the animals.

The otosilicate (biosilicate®) proved not ototoxic or vestibulotoxic for the guinea pigs tested, there was no destruction of inner or outer hair cells within 30 and 90 days of exposure, proving to be a material of good tolerability and compatibility as already shown in other studies^18^. In the study by Moura (2007) they also observed the formation of hydroxycarbonate apatite on the otosilicate surface after 24 hours, in cell culture liquid, showing its high bioactivity (class A). With this accelerated in vitro osteogenesis, there is a lower likelihood of extrusion of such material in the postoperative of ossiculoplasties.

Thus, by approval from the ethics committee of experimentation in humans, we started to use this material as middle ear prosthesis in humans, partial prosthesis - when we have the stapes or total prosthesis when we place the prosthesis directly on the stapes footplate. We started the surgical procedures in humans, expecting to be successful on the first results obtained; however, because of the reduced number of cases and the restricted follow up time, this study must be expanded.

## CONCLUSION

Otosilicate (biosilicate®) did not present ototoxicity or vestibulotoxicity for the lab animals studied, proving to be a safe and promising material to be used as a prosthesis for ossicular reconstruction in humans.
